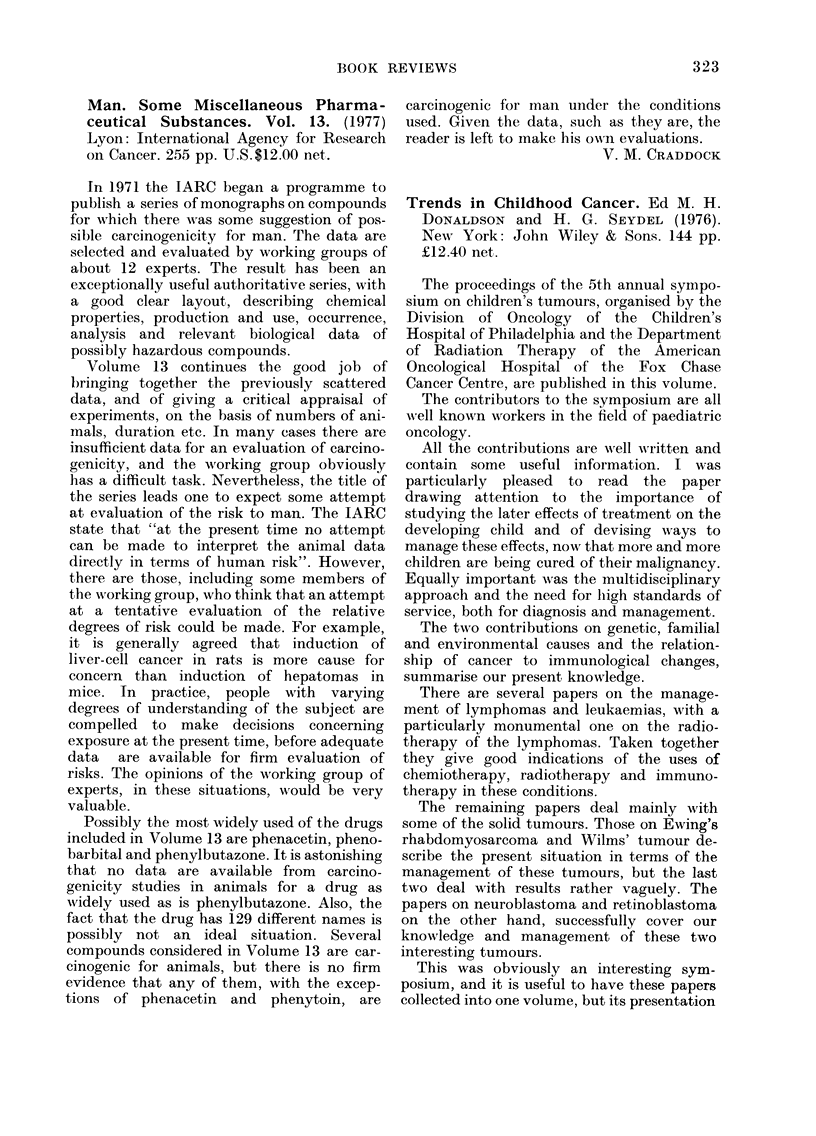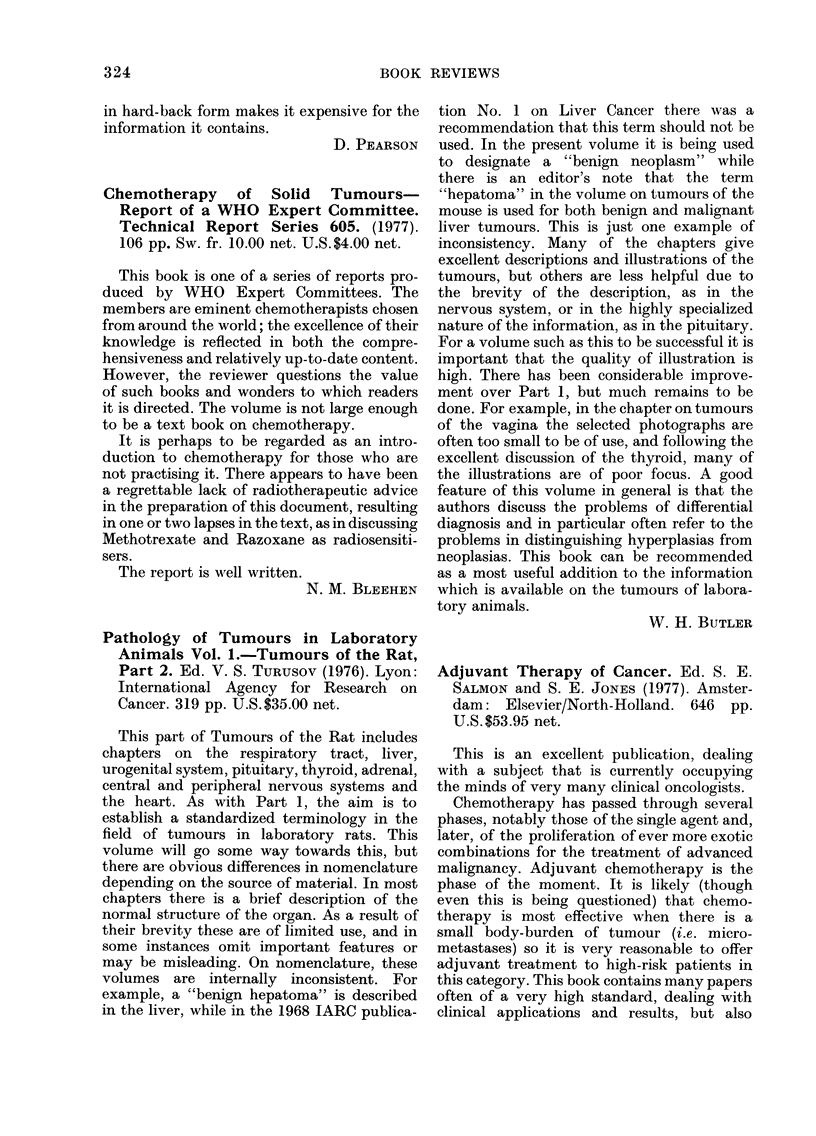# Trends in Childhood Cancer

**Published:** 1978-02

**Authors:** D. Pearson


**Trends in Childhood Cancer.** Ed M. H. DONALDSON and H. G. SEYDEL (1976). New York: John Wiley & Sons. 144 pp. £12.40 net.

The proceedings of the 5th annual symposium on children's tumours, organised by the Division of Oncology of the Children's Hospital of Philadelphia and the Department of Radiation Therapy of the American Oncological Hospital of the Fox Chase Cancer Centre, are published in this volume.

The contributors to the symposium are all well known workers in the field of paediatric oncology.

All the contributions are well written and contain some useful information. I was particularly pleased to read the paper drawing attention to the importance of studying the later effects of treatment on the developing child and of devising ways to manage these effects, now that more and more children are being cured of their malignancy. Equally important was the multidisciplinary approach and the need for high standards of service, both for diagnosis and management.

The two contributions on genetic, familial and environmental causes and the relationship of cancer to immunological changes, summarise our present knowledge.

There are several papers on the management of lymphomas and leukaemias, with a particularly monumental one on the radiotherapy of the lymphomas. Taken together they give good indications of the uses of chemiotherapy, radiotherapy and immunotherapy in these conditions.

The remaining papers deal mainly with some of the solid tumours. Those on Ewing's rhabdomyosarcoma and Wilms' tumour describe the present situation in terms of the management of these tumours, but the last two deal with results rather vaguely. The papers on neuroblastoma and retinoblastoma on the other hand, successfully cover our knowledge and management of these two interesting tumours.

This was obviously an interesting symposium, and it is useful to have these papers collected into one volume, but its presentation in hard-back form makes it expensive for the information it contains.

D. PEARSON